# A Suggestion

**Published:** 1880-11

**Authors:** 


### EDITORIAL.

### A SUGGESTION.

Some of the older members of our profession will remember that just before the war, at a meeting of the American Dental Convention, on motion of the lamented Dr Blakesby, a Committee of three was appointed to raise a fund, and secure therewith, a scientific member of our profession, who should devote his entire time to the investigation and development of dental science and such collateral subjects as would throw light on it. That committee consisted of Drs. A. Blakesly, J. Taft, and T. L. Buckingham. This committee issued circulars, as a step toward raising the fund, and secured the services of Dr. G. Watt, as the investigator and instructor. But one name besides Dr. Watt's was suggested to the committee, and that by a single individual, while scores recommended the selection of Dr. Watt.

The work was to begin with the Summer of 1861, but the cruel war defeated the whole thing. But now Dr. Watt is still with us and though not able for active practice, he is ready for the most active brain work, and his attainments in science are greatly in advance of what they were then. Why should not the various local and State Societies now secure his services in advancing the science of our beloved profession? A lecture from him on anaesthesia, the chemistry of the mouth; the nature, varieties, and treatment of dental caries, preventive as well as restorative; nutrition with reference to the teeth; and other subjects that might be selected. Such work would be of incalculable benefit to any society and to the profession.

His improvement in health restoring him fully for brain work, while his hands still lack their wonted skill, seems to indicate that his talents and attainments should not be lost to the profession. In order to introduce and test the feasibility of this suggestion, I would propose to the Executive Committee of the Ohio State Dental Society to make an effort to secure Dr. Watt's services, in the production of a lecture or paper, upon one or more of the subjects above indicated.

This, it seems to me, should be made a business matter. Dr. Watt is able now to render valuable service to the profession with his brain and pen, and if it is given a compensation should be rendered. Let the members of the society, who may see this suggestion and coincide with it, drop a line to Dr. R. G. Warner, Columbus, Chairman of the Executive Committee, in order that the committee may feel authorized to act in this matter. Something from our treasury, invested in this way, would be the means of doing much good in more directions than one. Dr. Watt has in former years given, gratuitously, to our profession far more brain work, than he has given to his own and his family's interest. Think of this, brethren, and let us bless ourselves, him, and the human race as well.

